# Gr1^+^ myeloid-derived suppressor cells participate in the regulation of lung–gut axis during mouse emphysema model

**DOI:** 10.1042/BSR20221041

**Published:** 2022-09-21

**Authors:** Jing Yang, Jiajia Zeng, Shuaini Yang, Xin Guan, Qiaoying Gao, Simeng He, Xiaoyang Wu, Lixiu Ge, Hong Bai

**Affiliations:** 1 Tianjin Key Laboratory of Acute Abdomen Disease Associated Organ Injury and ITCWM Repair, Institute of Acute Abdominal Diseases of Integrated Traditional Chinese and Western Medicine, Tianjin Nankai Hospital, Tianjin 300100, China; 2 Department of Immunology, Tianjin Key Laboratory of Cellular and Molecular Immunology, Key Laboratory of Immune Microenvironment and Disease (Ministry of Education), School of Basic Medical Sciences, Tianjin Medical University, Tianjin 300070, China; 3 Department of Anesthesiology and Critical Care Medicine, Tianjin Nankai Hospital, Nankai University, Tianjin 300071, China; 4 Department of Clinical Laboratory, Tianjin Nankai Hospital, Tianjin 300100, China

**Keywords:** COPD, Emphysema, lung-gut axis, macrophages, MDSCs

## Abstract

Background: Chronic obstructive pulmonary disease (COPD) is often accompanied by intestinal symptoms. Myeloid-derived suppressor cells (MDSCs) possess immunosuppressive ability in cancer, chronic inflammation, and infection. The aim of this study was to verify the distribution of MDSCs in emphysema mouse model and participation in lung–gut cross-talk.

Methods: Adult male C57BL/6 mice were exposed to cigarette smoke (CS) for 6 months or injected with porcine pancreas elastase to establish emphysema models. Flow cytometry and immunohistochemistry analysis revealed the distribution of MDSCs in tissues. The expression of inflammation and MDSCs-associated genes in the small intestine and colon were analyzed by real-time PCR.

Results: The small intestine and colon of CS-induced emphysematous mice displayed pathological changes, CD4^+^/CD8^+^ T cells imbalance, and increased neutrophils, monocytes, and macrophages infiltration. A significant expansion of MDSCs could be seen in CS-affected respiratory and gastrointestinal tract. Importantly, higher expression of MDSCs-related effector molecules inducible nitric oxide synthase (INOS), NADPH oxidase 2 (NOX2), and arginase 1 (ARG-1) suggested the immunosuppressive effect of migrated MDSCs (*P*<0.05).

Conclusion: These data provide evidence for lung–gut axis in emphysema model and the participants of MDSCs.

## Introduction

Chronic obstructive pulmonary disease (COPD) is a common chronic respiratory disease characterized by not fully reversible airflow limitation. It is usually induced by long-term exposure to toxic gases and particles from cigarette smoke (CS) and biomass fuel, causing impaired lung function, high morbidity, and mortality in patients worldwide [1,2]. Moreover, it has long been recognized that progressive inflammation in the airways, the alveoli, and the microvasculature is a primary feature of COPD [3]. Emphysema has been recognized as the main pathological component of COPD. It has always emphasized of research that focuses on the COPD pathogenesis with the characteristics of abnormal permanent enlargement of the airspaces and destruction of the alveolar wall [4]. However, the mechanisms that attack the initiation and progression of emphysema and decline of lung function are not well understood, consequently hampering the progression of effective therapy for COPD patients. Animal model of emphysema with pathological changes accurately recapitulating most of the key features of the human disease will be helpful in efforts to improve our understanding of COPD physiology, pathophysiology, and treatments [5].

Chronic airway diseases, like COPD, asthma, and respiratory virus infection, are often accompanied by gastrointestinal symptoms [6–8]. For example, associations between COPD and inflammatory bowel disease (IBD) have been demonstrated [9,10]. Epidemiologic studies have shown significant increases in incidence [11] and prevalence [12] of IBD in patients with airway diseases. Complementarily, many studies have highlighted a higher incidence of pulmonary inflammation in IBD, with IBD patients developing extraintestinal manifestations of diseases, such as pulmonary dysfunction [13]. Although the pathophysiologic mechanisms are still areas of intensive research, accumulating evidence suggest the influence of the gut condition on lung immunity, referred to as the lung–gut axis, which describes the common mucosal immune system of the respiratory and gastrointestinal tract.

Myeloid-derived suppressor cells (MDSCs) described a largely heterogeneous ensemble of myeloid cells with potent immunosuppressive activity in cancer and diseases including chronic inflammation, infection, autoimmune diseases, trauma, graft versus host disease, etc. [14]. This cell population was reported to be composed of granulocytes, monocytes, and other myeloid cells with distinct functional properties [15]. One of the primary functions of MDSCs is their capability to inhibit both the adaptive (CD8^+^ and CD4^+^ T lymphocytes) and innate immunity (natural killer [NK] cells) via the production of effector molecules, such as arginase 1 (ARG-1), nitric oxide, inducible nitric oxide synthase (INOS), and reactive oxygen species (ROS), etc. [16,17]. Poor lung function of COPD patients is thought to result, in part, from exaggerated innate immune-mediated pulmonary inflammation in response to chronic air pollutant exposure [18,19]. Furthermore, several lines of evidence suggest that emphysema pathogenesis involves the participation of a complex network of inflammatory cells, such as neutrophils, macrophages, and lymphocytes [20–22]. Based on the participants of these inflammatory cells in COPD pathogenesis and the heterogeneity of MDSCs, we hypothesize that MDSCs participate in the lung–gut cross-talk of mice model of pulmonary emphysema.

In the present study, we used mouse models of COPD, namely CS-induced and porcine pancreatic elastase (PPE)-induced emphysema, to examine the participation of MDSCs in the lung–gut axis of COPD pathogenesis. We observed apparent pathological changes, CD4^+^ T/CD8^+^ T cells imbalance, and increased innate immune cells (neutrophils, monocytes, and macrophages) in the small intestine and colon of emphysematous mice. Flow cytometry analysis found expansions of MDSCs to the respiratory and gastrointestinal tract. Importantly, higher expression of MDSC-related effector molecules in CS-affected intestinal tract, including INOS, NOX2, and ARG1, proves that migrated MDSCs played an immune regulatory role. Thus, our study recognized the existence of the lung–gut axis in the murine model of emphysema and demonstrated the regulatory role of MDSCs through modulating inflammatory levels.

## Materials and methods

### Mice

Male C57BL/6 mice at the age of 8–10-week-old were purchased from Beijing HFK Biotechnology Co. Ltd. (Beijing, China) and were housed in specific pathogen-free conditions under a 12-h light/dark cycle at 22–24°C each day.

For the whole-body CS exposure model, mice were exposed to CS according to a modified protocol from previously published work [23,24]. Briefly, whole-body exposure to CS (11 mg tar, 0.8 mg or less of nicotine, and 13 mg CO) occurred in an 18-L plastic chamber. Mice received smoke produced by burning filter-tipped ZuanShi cigarettes (China Tobacco Hebei Industrial Co., Ltd., China). Mice were exposed four times per day with a 30-min smoke-free interval, 5 days per week for 24 weeks at a concentration of 400–500 mg/m^3^ total particulates. Littermate control mice were exposed to normal air.

For PPE-induced emphysema mouse model, mice were anesthetized with a combined injection of xylazine (5 mg/kg) and ketamine (40 mg/kg), and then intratracheally injected with 4.8 U/mg of PPE (Cat#: E7885, Sigma, MO, USA) on day 0. Mice were sacrificed on day 21 after elastase administration.

All animal experiments were carried out at the Tianjin Nankai Hospital and followed the guidelines of the Animal Ethical and Welfare Committee and were approved by the Medicine Ethical Committee of Tianjin Nankai Hospital (number NKYY-DWLL-2020-117). At the end of the experiment, all animals were anesthetized by intraperitoneal pentobarbital sodium and sacrificed by CO_2_ asphyxiation.

### Histopathological examination

Lung tissue, small intestine, and colon were harvested aseptically from mice, fixed in 10% formalin for 24 h. After dehydration and embedding in paraffin, 4-μm serial sections were prepared for histological analysis and were stained with either hematoxylin-eosin (H&E) or periodic acid Schiff (PAS), strictly following the manufacturer’s instructions. Slides were reviewed in a blinded fashion and the degree of tissue damage was estimated through a light microscope. To measure alveolar destruction, airspace size was evaluated by quantifying the mean linear intercept (MLI) in the average of ten randomly selected fields per lung specimen from model or control mice (*n*=5), as previously reported [25].

### Cell isolation

Mesenteric lymph nodes (MLNs) and spleen single-cell suspensions were prepared by mechanical disruption and passed through a 40-µm cell strainer.

Lung was aseptically removed from mice, minced into small pieces by scissors, and incubated in a digestion medium containing 0.5 mg/ml collagenase IV (Sigma, MO, USA) in RPMI 1640 medium at 37°C for 30 min. After erythrocytes lysis, the cells were washed and kept on ice until labeling. The small intestine and colon were washed with cold PBS (1% BSA), cut into approximatively 1-cm long pieces, and then incubated for 20 min at 37°C in RPMI 1640 medium containing 5 mM EDTA with shaking. After three washing steps, the remaining tissue was digested for 50 min at 37°C with complete RPMI 1640 medium (10% FBS) containing 0.5 mg/ml collagenase IV and 1 mg/ml DNase I (Roche, Switzerland). Then, tissue suspension was passed through a 40-μm filter, and cells were collected and resuspended in PBS for further treatment.

### Flow cytometry

Single-cell suspensions (10^6^ cells in 100 µl total volume) were treated with antibodies to mouse CD16/32 pure antibody (BD Phamingen, CA, USA) for 30 min at 4°C in the dark. Then, the cells were incubated with indicated antimouse monoclonal antibodies for 30 min on ice in the dark for surface marker analysis. The following antimouse monoclonal antibodies were used: CD3, CD4, CD8, CD45, CD11b, CD86, CD206, Ly6C, Ly6G, Gr1, and F4/80, which were purchased from BioLegend (BioLegend, Inc., CA, USA). Isotype-matched antibodies were used as controls. All operations were performed strictly in accordance with protocols. Raw data were collected on an EXFLOW-206 flow cytometer (Dakewe Biotech Co. Ltd., Shanghai, China) and analyzed using FlowJo 10.0 software (BD, CA, USA).

### Immunohistochemistry

The 4-μm paraffin-embedded lung, small intestine, and colon sections were deparaffinized, rehydrated in xylene and an alcohol gradient, rinsed with distilled water and soak in PBS for 5 min and heated in a sodium citrate solution for antigen retrieval before immunohistochemical staining. The staining was performed strictly according to the Histostain – Plus Kits (Cat#: SP-0022, Bioss Antibodies). Briefly, endogenous peroxidase activity (3% hydrogen peroxide, 15–20 min) and nonspecific binding (goat serum, 15–20 min) were blocked, sections were incubated with the indicated primary antibody at 4°C overnight or at 37°C for 2–3 h in a humidified box, then washed three times with PBS and incubated with the secondary biotinylated IgG antibody at room temperature or 37°C for 15–20 min. Subsequently, incubated with Corseradase-labeled Streptamildew Lectein Working Solution (S-A/HRP) at room temperature or 37°C for 15–20 min. Finally, DAB or AEC reagent was used to detect these labeled antibodies. All images were detected using a microscope (Leica DMI4000B, Germany).

For immunohistochemistry, the expression of CD4, CD8, F4/80, and Gr1 in the small intestine and colon were detected with mouse monoclonal antibodies. Primary antibodies were CD4 (Cat#: 25229, Cell-Signaling Technologies, MA, USA), CD8 (Cat#: 98941, Cell-Signaling Technologies, MA, USA), F4/80 (Cat#: 123102, Biolegend), and Gr1 (Cat#: 108402, Biolegend). The secondary antibody is contained in the Histostain – Plus Kits.

### Quantitative real-time PCR

Total RNA was extracted from mouse small intestine samples and colon sections using the RNeasy Mini Kit (Qiagen, CA, USA). cDNA was synthesized by using PrimeScript RT reagent Kit (PRT) (Takara, Osaka, Japan). Real-time quantitative PCR (RT-qPCR) involved the use of TB Green Premix Ex Taq (Takara, Osaka, Japan) on a 7500 Real-Time PCR system (Applied Biosystems, MA, USA). All operations were performed strictly according to the manufacturer’s instructions. The fold changes in the gene expression levels of target genes were calculated with normalization to the endogenous control GAPDH values using the 2-∆∆Ct comparative cycle threshold method. All primer sequences used were as shown in Table 1.

**Table 1:** Primer sequences used for RT-qPCR analysis

Gene	Reverse sequence (5′-3′)	Forward sequence (5′-3′)
GAPDH	GGAAGAGTGGGAGTTGCTGTTG	CCTGGAGAAACCTGCCAAGTA
IFN-γ	TGACTCCTTTTCCGCTTCCTGAG	TGAACGCTACACACTGCATCTTGG
TNF-α	ACATTCGAGGCTCCAGTGAATTCGG	GGCAGGTCTACTTTGGAGTCATTGC
TGF-β	GGGGCTGATCCCGTTGATT	ACGTCACTGGAGTTGTACGG
IL-10	CTATGCAGTTGATGAAGATGTCAAA	ACCTGGTAGAAGTGATGCCCCAGGCA
ARG1	GTGATGCCCCAGATGGTTTTC	AACACGGCAGTGGCTTTAACCT
NOX2	TCATGGTGCACAGCAAAGTGAT	GACCCAGATGCAGGAAAGGAA
INOS	GAAACTATGGAGCACAGCCACAT	AGGAAGTGGGCCGAAGGAT
NQO1	AGTGCCCACAGAGAGGCCAAA	GCATTGGCCACACTCCACCAG
HO-1	GGCTGTCGATGTTCGGGAAGG	CACGCCAGCCACACAGCACTA
GCLC	CTCAAGAACATCGCCTCCATTCAG	ACATCTACCACGCAGTCAAGGACC
ZO-1	GAG CGG ACA AAT CCT CTC TG	GAA CGA GGC ATC ATC CCT AA
occludin	TCA TTC ACT TTG CCA TTG GA	TTT GTG GGA CAAGGA ACA CA

### Statistical analyses

All statistical analyses and preparation of graphs were performed with GraphPad Prism 5 software (GraphPad Software Ltd., CA, USA). A Student’s *t*-test was used for a two-group comparison. Statistical differences among multiple groups were assessed with one-way ANOVA, followed by the Tukey’s test. Error bars in the data are presented as the means ± SD, and *P*-values<0.05 were regarded as statistically significant.

## Results

### Chronic whole-body CS exposure or PPE intratracheal instillation results in lung emphysema

In the present study, two mice models of experimental emphysema were established by chronic whole-body exposure to CS for 24 weeks or intratracheal administration of PPE for 21 days.

Compared with the control mice, CS exposure and PPE administration groups displayed typical manifestations of emphysema. As shown in Supplementary Figure S1A,B, both emphysema models exhibited dramatically airway space enlargements and increased mean linear intercepts (MLI) of lung tissue, with increased destruction of the alveolar wall and the rupture of the alveolar septum (MLI: air exposed control group: 33.41±4.47 μm; smoke group: 67.86±15.09 μm; PPE group: 69.35±16.02 μm). Further PAS staining showed that CS exposure and PPE administration induced more epithelial cells in lung tissues (PAS positive nuclei numbers: air exposed control group: 14.58±0.53; smoke group: 19.88±0.84; PPE group: 18.10±2.10, Supplementary Figure S1C,D). These results suggest that the pulmonary emphysema mouse model was successfully established.

### Mouse model of emphysema displayed intestinal pathological changes

**Figure 1:**
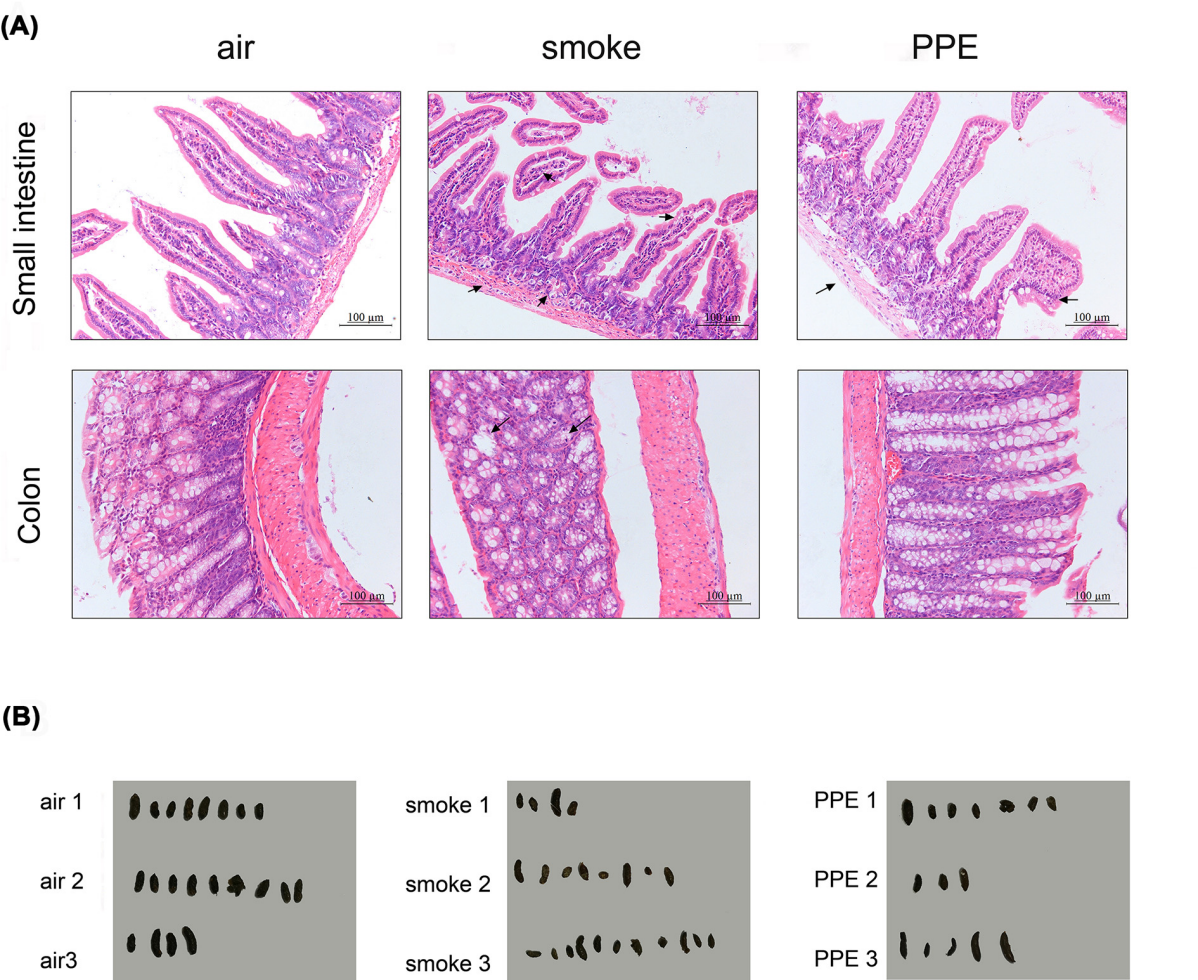
Mice model of emphysema displayed intestinal pathological changes (**A**) Comparison of H&E staining of the small intestine (upper, Scale bar = 100 μm) and colon (lower, Scale bar = 100 μm) from air-exposed control and emphysema model mice were performed (*n*=5 per group). (**B**) Effects of CS and PPE on the feces of C57BL/6 mice. Feces collected from air-exposed control and emphysema model mice under the normal diet for 1 h were represented (*n*=3 per group).

To determine whether lung inflammations can affect the intestines, we collected intestinal samples from our well-established experimental emphysema mouse models. Compared with air-exposed mice, H&E results presented pathological changes in the small intestine and colon sections, especially in the small intestine, including inflammatory cell infiltration and epithelial architecture destruction (Figure 1A). Furthermore, irregular stool specimens were observed from CS exposure and PPE-induced emphysema mice, suggesting intestinal dysfunction in emphysema models (Figure 1B).

### Mice model of emphysema showed T-cell immune dysregulation in the respiratory and intestinal tract

**Figure 2:**
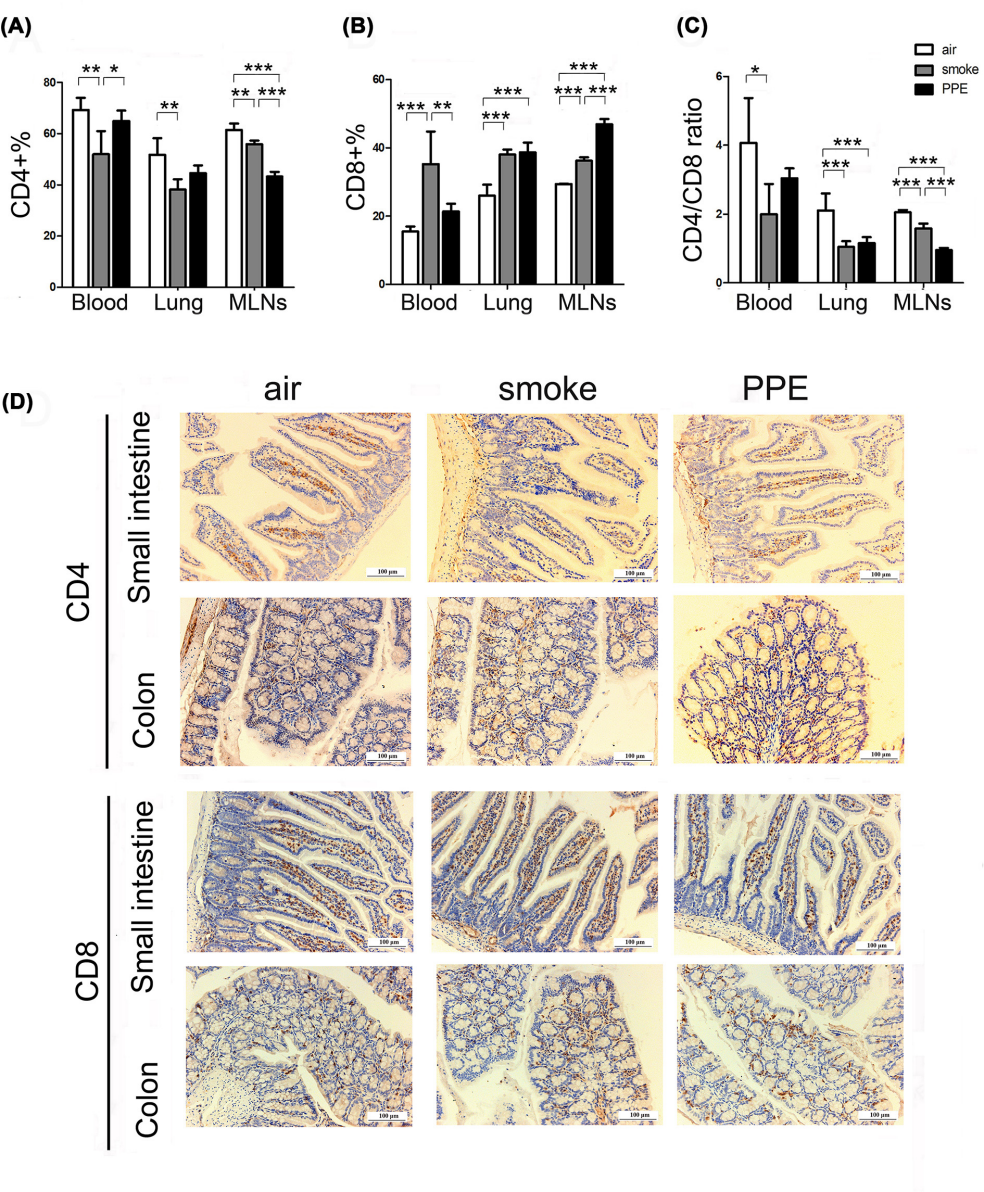
Changes in CD4^+^ T cells and CD8^+^ T cells occurred in the emphysema model (**A–C**) The percentage of CD3^+^CD4^+^ and CD3^+^CD8^+^ T cells, as well as the CD4^+^/CD8^+^ ratio in the blood, lung, and MLNs of air-exposed control (white histograms), CS exposure (gray histograms), and PPE administration (black histograms) mice were analyzed by flow cytometry as described (*n*=5 mice per group). Data are represented as means ± SD by one-way ANOVA. *P<0.05, **P<0.01, ***P<0.001. (**D**) Representative Immunohistochemical staining images of CD4^+^ and CD8^+^ T cells in the small intestine and colon from air-exposed control or emphysema model mice. Scale bar = 100 μm.

To assess the immunologic functions of emphysema-affected organs, we compared the numbers of CD4^+^ and CD8^+^ T-cell populations in the blood, lung, and MLNs of air-exposed and pulmonary emphysema mice via flow cytometry analysis first. Compared with normal air-exposed WT controls, exposure to CS resulted in a significantly decrease of CD4^+^ T cells (Blood: air-exposed control group: 69.18±4.79%; smoke group: 51.93±9.06; Lung: air-exposed control group: 51.69±6.48%; smoke group: 38.16±4.05; MLNs: air-exposed control group: 61.47±2.45%; smoke group: 55.84±1.40, Figure 2A) and increase of CD8^+^ T cells (Blood: air-exposed control group: 15.51±6.48%; smoke group: 35.19±9.62; Lung: air-exposed control group: 26.01±3.21%; smoke group: 38.08±1.45; MLNs: air-exposed control group: 29.36±0.15%; smoke group: 36.22±0.99, Figure 2B), as well as dramatically down-regulated CD4/CD8 ratio (Blood: air-exposed control group: 4.07±1.31%; smoke group: 1.99±0.88; Lung: air-exposed control group: 2.11±0.49%; smoke group: 1.05±0.16; MLNs: air-exposed control group: 2.05±0.07%; smoke group: 1.58±0.14, Figure 2C) in the blood, lungs, and MLNs tissues. Similarly, we also found the same tendency of T-cell subsets imbalance in PPE-induced emphysema group. The T-cell immune responses disorders in the intestinal tract were further supported by immunohistochemistry analysis (Figure 2D), wherein fewer CD4-positive cells and more CD8-positive cells were found both in the small intestine and colon of emphysema mice. These results suggested that pulmonary emphysema seems to lead to T-cell immune dysregulation and subsequent immunological abnormalities in the gut.

### Mice model of emphysema displayed intestinal inflammation

**Figure 3:**
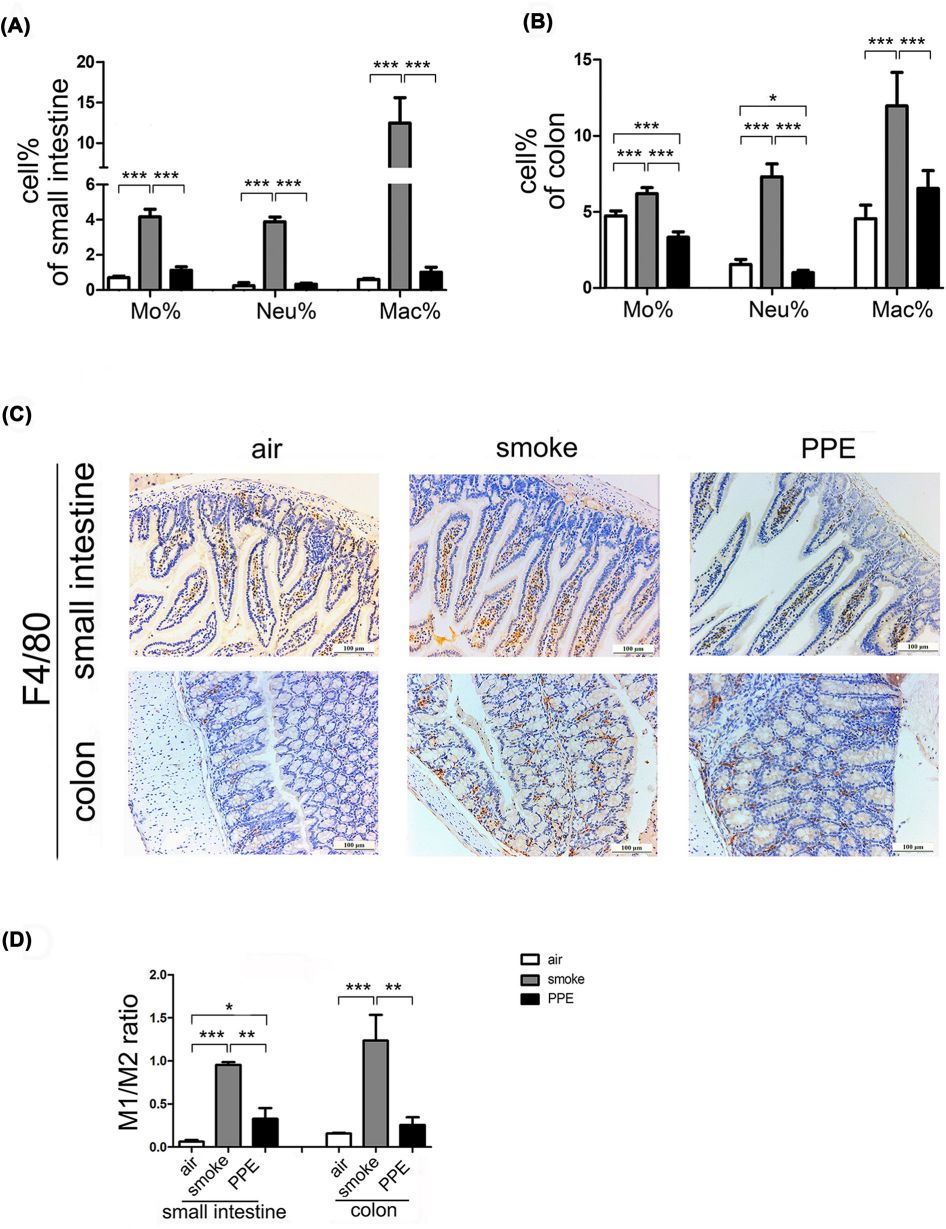
Monocytes, neutrophils, and macrophages increased in chronic CS-treatment emphysema model mice Flow cytometry was used to measure the percentage of CD11b^+^ Ly6C+ cells (‘inflammatory’ monocytes), CD11b^+^ Ly6G+ cells (neutrophils), and CD11b^+^ F4/80^+^ cells (macrophages) in the small intestine (**A**) and colon (**B**) of air-exposed control and emphysema model mice. (**C**) Immunohistochemical evaluation of F4/80 expression in the small intestine (upper) and colon (lower) from air-exposed control or emphysema model mice (*n*=5 per group). Scale bar = 100 μm. (**D**) The ratio of M1 (CD11b^+^F4/80^+^CD86^+^ cells) to M2 (CD11b^+^F4/80^+^CD206^+^ cells) were assessed in the small intestine and colon from three groups (*n*=5).

COPD is characterized by persistent airflow limitation and increased airway inflammation [26]. We next assessed the inflammatory cells in the emphysema-affected intestine and colon. Flow cytometry (Supplementary Figure S2) and analysis (Figure 3A,B) revealed higher percentages of ‘inflammatory’ monocytes (CD11b^+^ Ly6C^+^) and neutrophils (CD11b^+^ Ly6G^+^) both in the small intestine and colon of model of emphysema in mice induced by long-term CS exposure compared with control group exposed to normal air (*P*<0.001). In contrast, PPE administration dramatically decreased the number of monocytes and neutrophils in colons without significant changes in small intestine tissues than in the air-treated group. This might be associated with occludin overexpression and enhancement of epithelial barrier function (Supplementary Figure S3B).

Macrophages exhibit considerable diversity and plasticity, enabling them to respond effectively to the stimulation of environmental signals by changing their phenotypes to classically activated macrophages (M1) or alternatively activated macrophages (M2), posing proinflammatory or immunoregulatory functions, respectively [27]. As shown in Figure 3A,B, flow cytometry analysis reveals the accumulation of macrophages (CD11b^+^ F4/80^+^) in CS-treated mice compared with in control mice, which then verified by immunohistochemical evaluation (Figure 3C). In addition, a significantly upregulation of M1/M2 ratio (CD86^+^/CD206^+^) in the small intestine and colon of CS-exposed mice suggested the proinflammatory status of intestinal macrophage (Figure 3D). Interestingly, PPE administration drove a shift of M1 polarization in the small intestine and colon, although the number of total macrophages in that was comparable between air- and PPE-treated groups.

**Figure 4:**
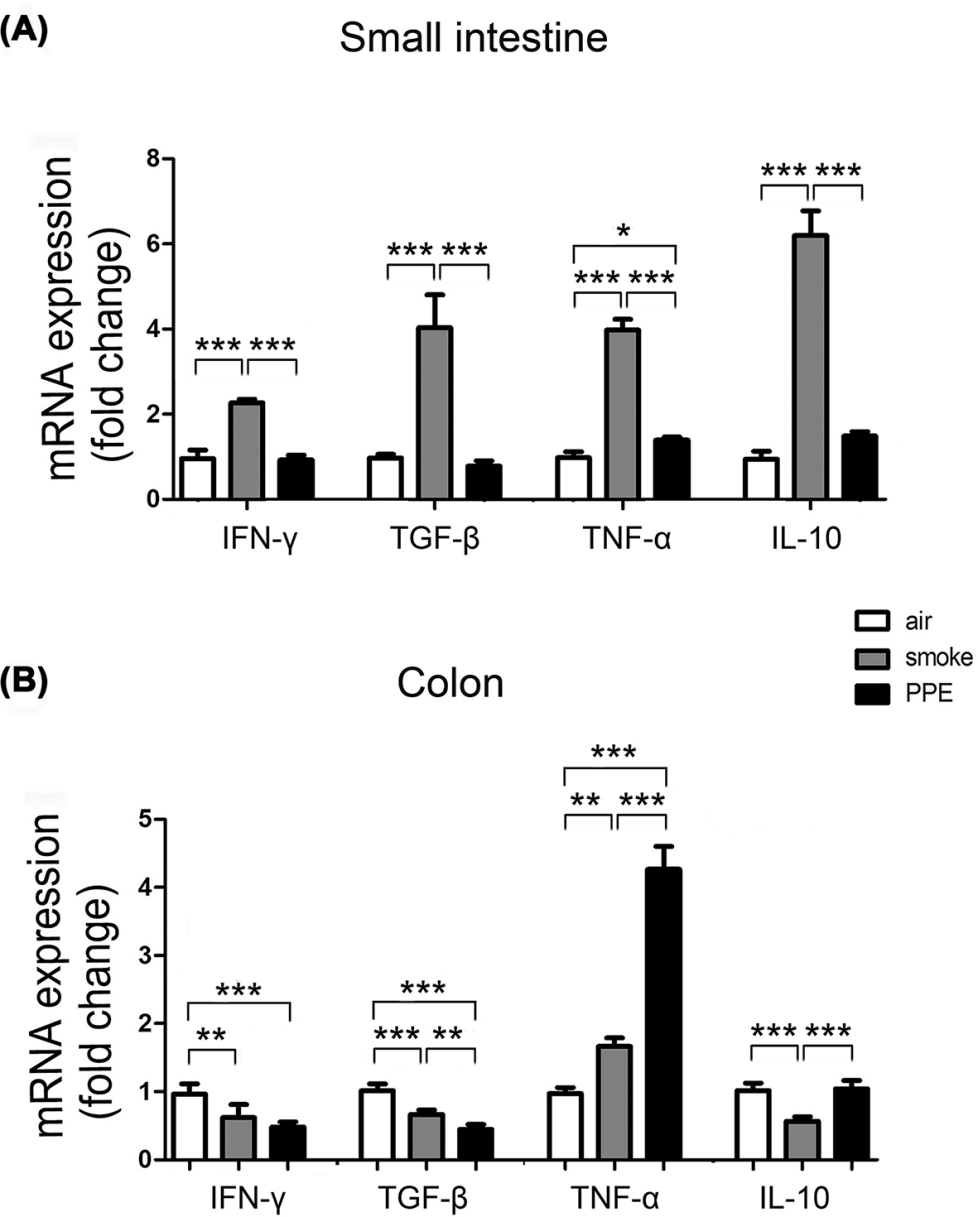
Intestinal inflammation increased in emphysema model mice Total RNA extracted from small intestine (**A**) and colon (**B**) of air-exposed control or emphysema model mice was assayed for IFN-γ, TNF-α (proinflammatory cytokines), TGF-β, and IL-10 (anti-inflammatory cytokines) mRNA expression by qPCR using specific primers. Data are represented as means ± SD by one-way ANOVA. *P<0.05, **P<0.01, ***P<0.001.

Given that CS-exposed mice had increased intestinal inflammatory cells, we next assessed the mRNA expression of inflammatory cytokines in the small intestine and colon. PCR results showed higher mRNA expression of IFN-γ (smoke group: 2.26-fold higher; *P*<0.001) and TNF-α (smoke group: 3.98-fold higher; *P*<0.001) as well as higher anti-inflammatory factor TGF-β (smoke group: 4.03-fold higher; *P*<0.001) and IL-10 (smoke group: 6.19-fold higher; *P*<0.001) in the small intestine of CS exposure (Figure 4A), wherein decreased IFN-γ (smoke group: 0.62-fold higher; *P*<0.01), TGF-β (smoke group: 0.66-fold higher; *P*<0.001) and IL-10 (smoke group: 0.57-fold higher; *P*<0.001) but increased TNF-α in the colon (smoke group: 1.66-fold, 0.62-fold higher; *P*<0.01; Figure 4B). Notably, the expression of inflammation-related genes TNF-a was up-regulated in the colon tissues of PPE-treated mice compared with the control and CS-exposed groups. These data suggest that pulmonary emphysema plays a role in inducing intestinal inflammation dysfunction and has different impacts on the small intestine and colon.

### Migration of MDSCs to the respiratory and gastrointestinal tract was elevated in mice model of CS-induced emphysema

**Figure 5:**
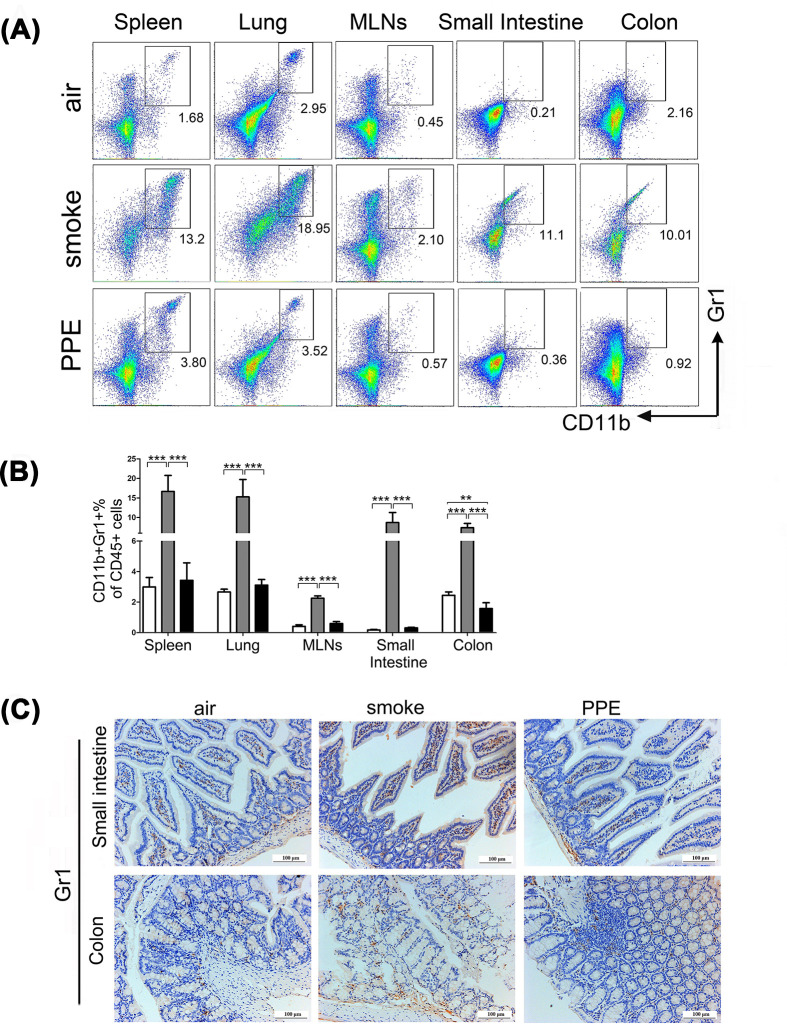
Chronic CS treatment increased the expansion of CD11b^+^Gr1^+^ MDSCs cells in tissues (**A,B**) The percentage of CD11b^+^Gr1^+^ MDSCs in the spleen, lung, MLNs, small intestine, and colon of air-exposed control and emphysema model mice was quantified by flow cytometry (*n*=5). Data are represented as means ± SD by one-way ANOVA. **P<0.01, ***P<0.001. (**C**) Immunohistochemical evaluation of Gr1 expression in the small intestine (upper) and colon (lower) from air-exposed control or emphysema model mice. Small intestine (upper) and colon (lower) staining with Gr1 marker (brown).

MDSCs were previously shown to have the potential to suppress immune cells (especially T lymphocytes) as a regulatory mechanism. Hence, we investigated the expansion of CD45^+^CD11b^+^Gr1^+^ MDSCs in the spleen, lung, MLNs, small intestine, and colon between the air-exposed control group and mice with emphysema combining flow cytometry and immunohistochemistry analyses. As shown in Figure 5A,B, flow cytometry analysis identified increased percentages of MDSCs in these tissues (Spleen: air-exposed control group: 2.99±0.62%; smoke group: 16.64±4.10%; PPE group: 3.43±1.15%; Lung: air-exposed control group: 2.66±0.18%; smoke group: 15.26±4.45%; PPE group: 3.11±0.37%; MLNs: air-exposed control group: 0.41±0.10%; smoke group: 2.25±0.15%; PPE group: 0.60±0.12%; Small intestine: air-exposed control group: 0.17±0.03%; smoke group: 8.69±2.57%; PPE group: 0.31±0.03%; Colon: air-exposed control group: 2.45±0.22%; smoke group: 7.36±1.13%; PPE group: 1.57±0.38%) of model mice as compared with the control group. Consistent with these data, immunohistochemistry analysis verified further that the small intestine and colon sections of emphysema animals were surrounded by increased Gr1^+^ MDSC-positive cells compared with wild-type mice (Figure 5C).

### Higher expression of MDSCs-related effector molecules was identified in the intestinal tract of mouse model of CS-induced emphysema

**Figure 6:**
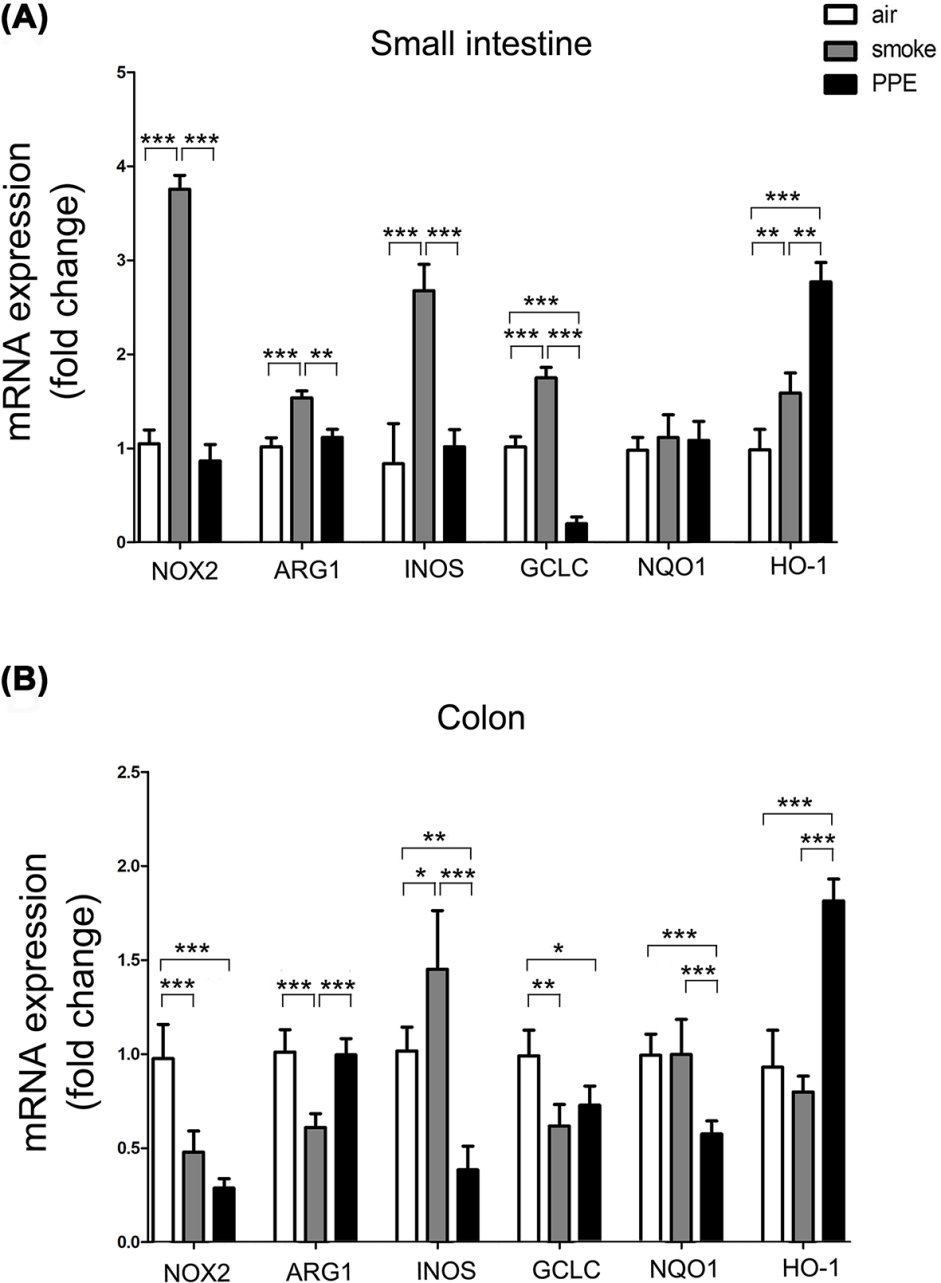
Chronic CS treatment affects the expression of MDSCs-related inhibitory genes in the intestine Total RNA extracted from small intestine (**A**) and colon (**B**) of air-exposed control or emphysema model mice. Quantification and statistical analysis results of NOX2, ARG1, and INOS, glutamic acid cysteine ligase catalytic subunit (GCLC), NQO1, and HO-1 mRNA expression by qPCR using specific primers in air-exposed control (white histograms), CS exposure (gray histograms), and PPE administration (black histograms) mice (*n*=5). Data are represented as means ± SD by one-way ANOVA. *P<0.05, **P<0.01, ***P<0.001.

To test the biological functions of migrated MDSCs in the emphysema-induced lung–gut cross-talk, PCR analysis was conducted to clarify the expression of NADPH oxidase 2 (NOX2), enzyme ARG1, and INOS in the intestinal tract, which are reported to be produced in MDSCs and featured by this immune subset to exert their immunosuppressive function [28]. CS-induced emphysema mice showed increased NOX2 (smoke group: 3.76-fold higher; *P*<0.001), ARG1 (smoke group: 1.54-fold higher; *P*<0.001), and INOS (smoke group: 2.68-fold higher; *P*<0.001) expression than the control group in the small intestine (Figure 6A); however, decreased NOX2 (smoke group: 0.48-fold higher; *P*<0.001) and ARG1 (smoke group: 0.61-fold higher; *P*<0.001) in the colon (Figure 6B). As for PPE model mice, expression of NOX2 and INOS gene was decreased in the colon, whereas no significant differences be detected in the small intestine of the expression of NOX2, ARG1, and INOS gene when comparing PPE model and control mice, as measured by qPCR.

Oxidative stress was reported as an important mechanism for the occurrence and exacerbation of COPD [4,29]. We next detected the expression level of oxidative stress-related proteins heme oxygenase 1 (HO-1), GCLC, and NAD(P)H quinone oxidoreductase-1 (NQO-1). The PCR results showed significant differences between the emphysema and control mice, especially in the PPE-induced pulmonary emphysema mice model, acting as further evidence for the lung–gut cross-talk in this model.

## Discussion

Both respiratory and gastrointestinal tracts belong to mucosal tissues. Recent research on the early embryo, signal pathway, mucosal immunity, and gut microbiota on lung immunity further proposed the correlation between the lung and gut [30]. With the same embryonic origin and structural similarities, the respiratory and gastrointestinal tracts have different environments and functions in mature individuals [31]. Under pathological statements, these commonalities in structures may account for an overlap of pathogenic risk factors and comparable immune responses between the gut–lung axis. Many mechanisms could participate in this pathologic process, such as gut microbiota alternation, transportation of microRNAs and inflammasomes, and metabolites [32–34]. Rutten et al. reported increased gut permeability in COPD patients [35]. Further studies identified that intestinal barrier dysfunction would be driven by systemic hypoxia induced by the impaired pulmonary gas exchange during chronic CS exposure in animal models and patients [35,36].

In line with the strong epidemiological evidence that a vital cross-talk between the gut and lungs [26,37], we demonstrated here that intestinal dysfunction occurred in the emphysema model as CS exposure and PPE-induced emphysema mice with irregular stool shapes and intestinal pathological changes compared with air-exposed control mice. Smoking is a common risk factor not only for the development of COPD but also for a number of other diseases. It is well established that cigarette smoking directly drives lung inflammatory response; however, effect on other tissues is incompletely understood. Our study here demonstrated the influence of emphysema on the intestinal tract in CS-induced mouse model. First, both the small intestine and colon displayed severe pathology, imbalanced CD4^+^/CD8^+^ T-cell ratio and inflammation. Furthermore, we found the accumulation of MDSCs and changes of MDSC-related effector molecules in lung–gut axis-involved tissues. The present study identified the disorder of the immune system and inflammatory response in the intestinal tract of emphysema mice model and revealed the unappreciated participation of MDSCs in COPD pathogenesis, following long-term CS exposure. These results provide experimental evidence for the existence of the lung–gut axis in COPD using the CS exposure-induced COPD mouse model.

Myeloid cells represent a highly diverse population comprising mononuclear cells and granulocytic cells [38]. Myelopoiesis against pathogenic signals is a critical protection mechanism for the host. However, the persistent stimulation associated with chronic infection, inflammation may induce modest but persistent myelopoiesis. Myeloid cells generated under these conditions take on altered biochemical profiles and functional activity compared with neutrophils and monocytes. The main functional characteristic of these abnormally differentiated cells, referred to as MDSC, is their potent ability to suppress various immune responses [14,39]. Based on this characteristic, one of the major functional roles of MDSCs is suppressing antitumor immunity [40–42]. MDSCs also suppress inflammation and promote insulin sensitivity in obesity [43]. It was reported that exposure of mice to CS causes the accumulation of this group of cells in the lungs and spleens [44]. In the present study, we further identified the migration of MDSCs in the lung and gut of CS exposure-induced emphysematous mice, which expanded the extensive regulation of MDSCs in lung disease.

Many mechanisms could participate in the gut–lung cross-talking process in COPD models, such as gut microbiota alternation, transportation of microRNAs and inflammasomes, and metabolites [32–34]. Here, we successfully adopt two groups of emphysema mouse models by CS chronic exposure and PPE administration, respectively. However, changes in the gastrointestinal tract were not wholly the same in the two groups, especially in intestinal inflammation, MDSCs accumulation, and oxidative stress responses. Research indicated that long-term CS exposure resulted in intestinal mucosal barrier dysfunction in a rat model of COPD [45]. Rutten et al. also reported increased gut permeability in COPD patients [35]. Further studies identified intestinal barrier dysfunction driven by systemic hypoxia induced by impaired pulmonary gas exchange during chronic CS exposure in animal models and patients [35,36]. Similarly, we demonstrated that the mRNA levels of the tight junction proteins zona occludens-1 (ZO-1) and occludin decreased in the small intestine and colon tissues of smoke mice compared with those of control groups. However, reduced ZO-1 and occludin expression in the small intestine but increased occludin level in the colon tissues could be seen in PPE-induced mice compared with control mice, which might be responsible for the impaired accumulation of monocytes, neutrophils, and Gr1^+^ MDSCs into the colon in this model. As to MDSCs level, the PPE group showed inapparent MDSCs accumulation, lower MDSCs-related inhibitory genes changes, and more tremendous oxidative stress-related molecule changes than smoke-induced emphysema. It seems that PPE models of emphysema do not trigger all of the physiological events that CS models do [46], proving that the CS-induced mice model appears to best represent human emphysema’s pathogenesis, including impaired lung function, emphysema, small airway remodeling, chronic lung inflammation, and pulmonary hypertension.

Notably, inflammatory responses were different in the small intestine and colon of emphysema mice models in our study, which indicated that the influence of lung disease on the gastrointestinal tract was dependent on the location. In the CS-exposure mouse model, the percentages of proinflammatory cells and proinflammatory cytokines genes (IFN-γ, TNF-α) were significantly higher in the small intestine than colon tissues. Correspondingly, MDSCs and anti-inflammatory genes (TGF-β, IL-10) were also up-regulated. These results suggest that proinflammatory and anti-inflammatory responses might reach balance at a higher level for the presence of gut-associated lymphoid tissue (GALT) in the small intestine. In contrast, both anti-inflammatory and MDSCs functional-related genes (NOX2 and ARG1) expression levels decreased significantly in colon tissues of CS exposure mice, suggesting the downregulated immunosuppressive role of migrated MDSC in the colon. Similar to ours, it is reported cigarette smoking has a diverse effect on the colon and ileum. In a smoking cessation mice model, cessation led to pathological amelioration to different extents in the colon and ileum, which may be due to the differences in microbiota and basal oxygen tensions [47,48].

In conclusion, our study demonstrated that MDSCs participate in the lung–gut axis in the mouse model of COPD. Though future experiments are needed to explore the potential molecular mechanism and biological effect of this group of immunosuppressive cells, our study provides sufficient experimental evidence for the lung–gut axis in the emphysema model. More importantly, our findings reveal a new biological application of MDSCs, promoting the in-depth understanding of the of COPD pathogenesis, will provide new strategies for research and potential immunotherapy targets for COPD.

## Supplementary Material

Supplementary Figures S1-S3

## Data Availability

The data used to support the findings of the present study are available from the corresponding author upon request.

## Abbreviations

AEC: 3-amino-9-ethylcarbazole
ALI: average linear intercept
ANOVA: analysis of variance
ARG1: enzyme arginase 1
BSA: bovine serum albumin
COPD: chronic obstructive pulmonary disease
CS: cigarette smoke
DAB: diaminobenzidine
EDTA: ethylene diamine tetraacetic aci
FBS: fatal bovine serun
GALT: gut-associated lymphoid tissue
GCLC: Glutamate-Cysteine Ligase Catalytic Subunit
HO-1: heme oxygenase 1
H&E: hematoxylin-eosin
IBD: inflammatory bowel disease
INOS: inducible nitric oxide synthase
MDSC: myeloid-derived suppressor cell
MLI: mean linear intercept
MLN: mesenteric lymph node
M1: classically activated macrophages
M2: alternatively activated macrophages
NK: natural killer
NOX2: NADPH oxidase 2
NQO-1: quinone oxidoreductase-1
PAS: periodic acid schiff
PBS: phosphate balanced solution
PPE: porcine pancreatic elastase
qPCR: quantitative PCR
ROS: reactive oxygen species
RPMI: Roswell Park Memorial Institute
SD: standard deviation
WT: wild type
ZO-1: zona occludens-1
