# Supplementary material for: Gr1+ myeloid-derived suppressor cells participate in the regulation of lung–gut axis during mouse emphysema model
Source: Biosci Rep. 2022 Sep 21;42(9):BSR20221041. doi: 10.1042/BSR20221041 (PMC9508528; doi:10.1042/BSR20221041)
Supplement: Supplementary Figures S1-S3 [file BSR-2022-1041_supp.pdf]

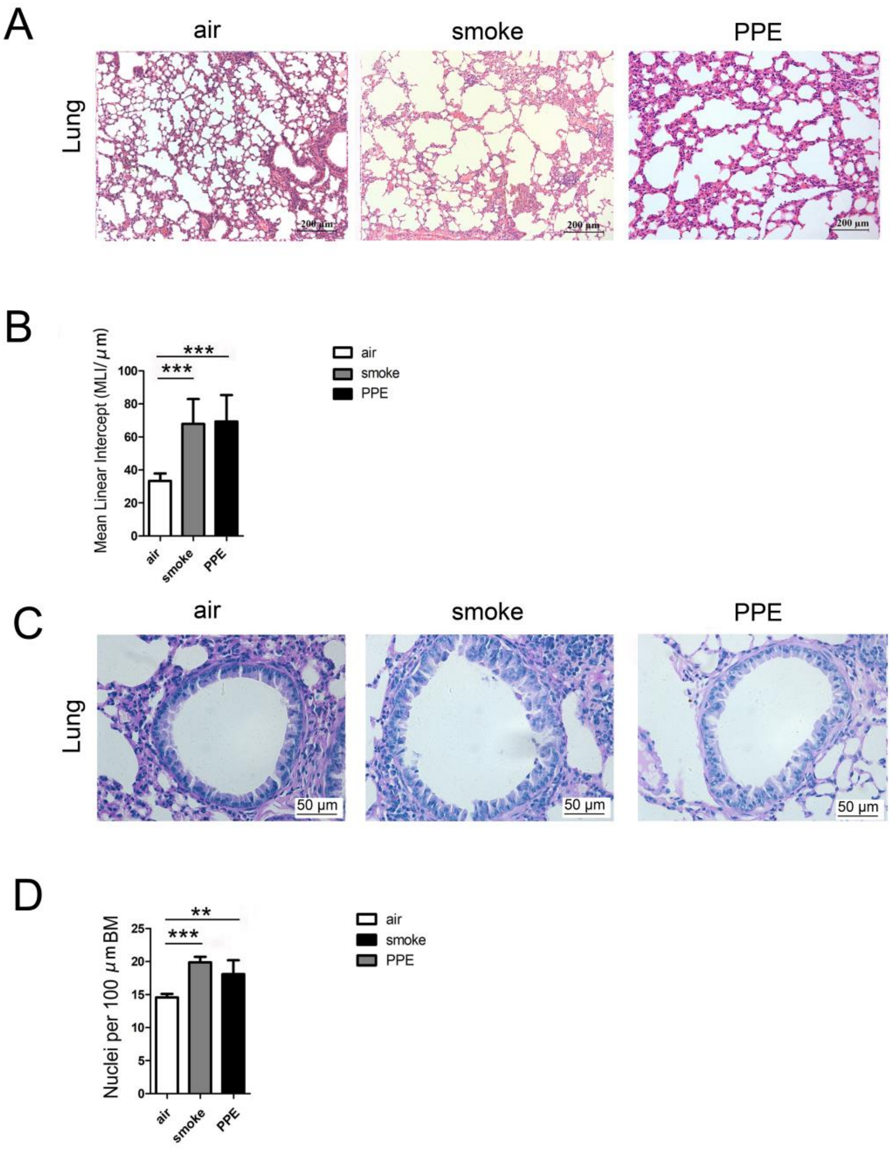
A
air
smoke
PPE
Lung
200 μm
B
Mean Linear Intercept (MLI/μm)
***
***
0
20
40
60
80
100
air
smoke
PPE
C
Lung
50 μm
D
Nuclei per 100 μm BM
**
***
0
5
10
15
20
25
air
smoke
PPE

Figure S1. Chronic whole-body CS exposure or PPE intratracheal instillation results in lung emphysema. Experimental emphysema models were induced by chronic whole-body exposure to cigarette smoke (CS) for 24 weeks or intratracheal administration of porcine pancreatic elastase (PPE) for 21 days. **(A)** A comparison of Hematoxylin and eosin (H&E) staining of the lung from air-exposed control mice and emphysema model mice was performed. (Scale bar, 200μm). **(B)** Quantification of mean linear intercept (MLI) in alveoli of air-exposed control (white histograms), CS exposure (gray histograms) and PPE administration (black histograms) mice (n=5 per group). **(C-D)** Formalin-fixed lung tissue was acid-Schiff (PAS) stained and the number of epithelial cells was evaluated. (Scale bar = 50μm).

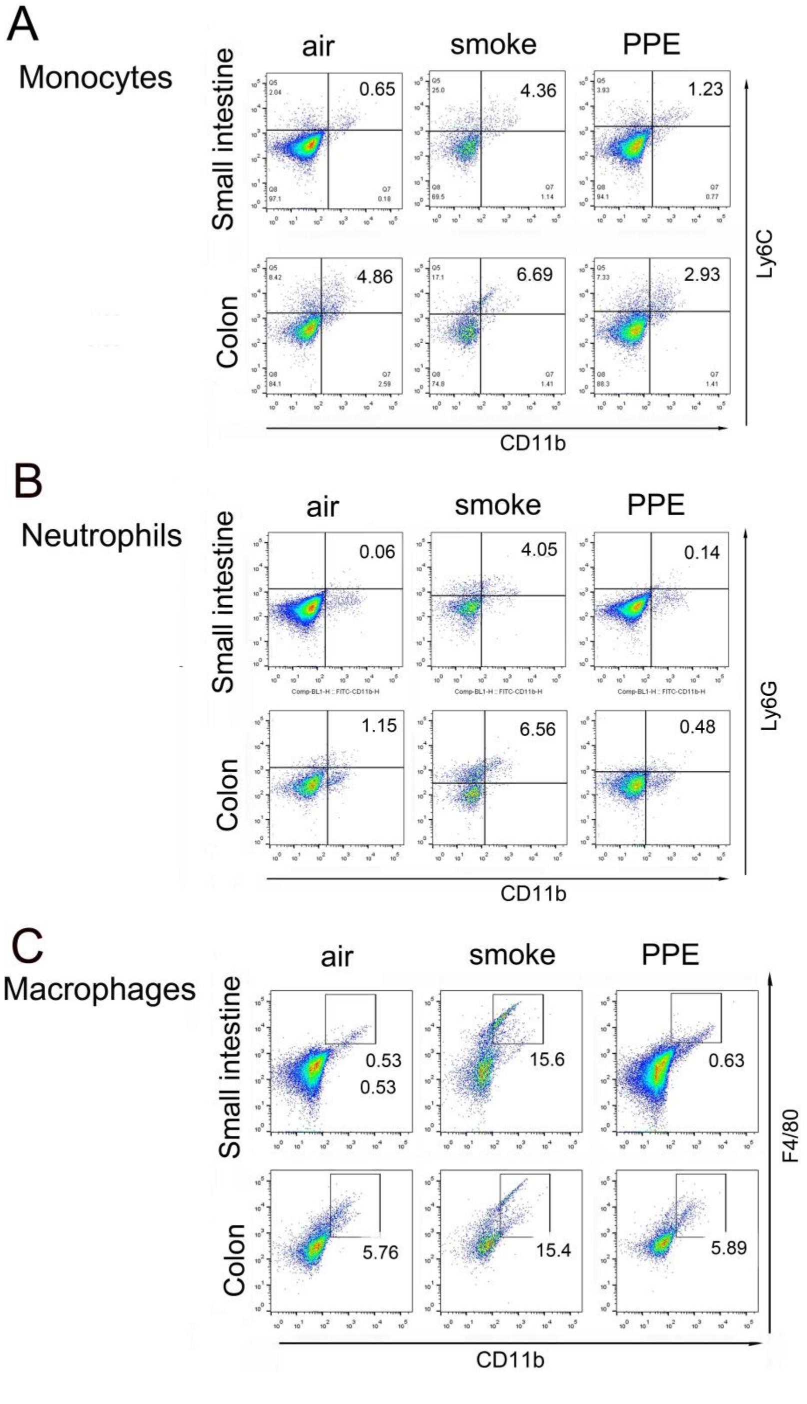
A
Monocytes
air
smoke
PPE
Small intestine
Colon
0.65
4.36
1.23
4.86
6.69
2.93
Ly6C
CD11b
B
Neutrophils
air
smoke
PPE
Small intestine
Colon
0.06
4.05
0.14
1.15
6.56
0.48
Ly6G
CD11b
C
Macrophages
air
smoke
PPE
Small intestine
Colon
0.53
0.53
15.6
0.63
5.76
15.4
5.89
F4/80
CD11b

Figure S2. Representative dot plots from flow cytometric analyses. The percentage of $CD11b^+Ly6C^+$ monocytes **(A)**, $CD11b^+Ly6G^+$ neutrophils **(B)** and $CD11b^+F4/80^+$ macrophages **(C)** were measured by flow cytometry in the small intestine and colon of air-exposed control and emphysema model mice.

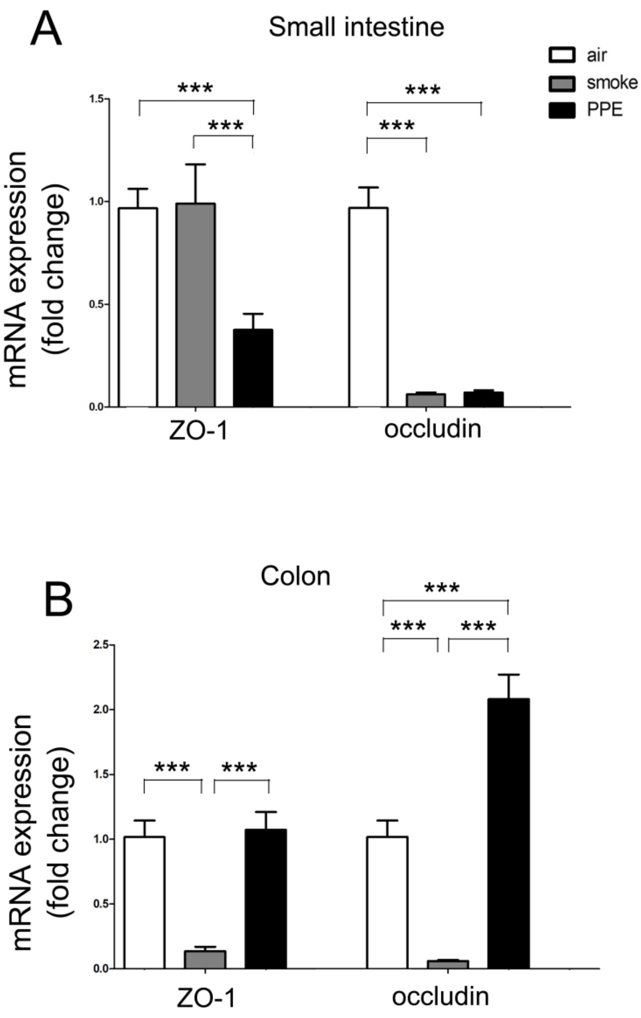
A
Small intestine
air
smoke
PPE
***
***
***
***
mRNA expression
(fold change)
1.5
1.0
0.5
0.0
ZO-1
occludin
B
Colon
***
***
***
***
***
mRNA expression
(fold change)
2.5
2.0
1.5
1.0
0.5
0.0
ZO-1
occludin

Figure S3. Chronic cigarette smoke and PPE treatment affects the expression of endothelial tight junction proteins ZO-1 and occludin. qRT-PCR was used to measure the transcripts of ZO-1 and occludin in the small intestine **(A)** and colon **(B)** of air-exposed control (white histograms), CS exposure (gray histograms) and PPE administration (black histograms) mice (n=5). Data are represented as means ± SD by one-way ANOVA. *P < 0.05, **P < 0.01, ***P < 0.001.
